# Evaluation of an International Point of Care Ultrasound (POCUS) Training Program for Internal Medicine Physicians

**DOI:** 10.24908/pocusj.v10i01.18429

**Published:** 2025-04-15

**Authors:** Katherine Otto Chebly, Elizabeth Hernández, Mary Cifelli, Michael Janjigian

**Affiliations:** 1 Division of General Internal Medicine and Clinical Innovation, Department of Medicine, NYU Grossman School of Medicine, New York, NY, USA; 2 Facultad de Medicina, Universidad Central de Venezuela, Ciudad Universitaria, Caracas, VEN

**Keywords:** international POCUS, global health, medical education, health equity, Venezuela

## Abstract

**Background:**

Point of care ultrasound (POCUS) training in internal medicine (IM) training remains largely unavailable in lower-resourced health systems globally. Longitudinal inter-institutional collaboration, based in health equity principles, offers a potential mechanism for more accessible and effective IM POCUS education.

**Methods:**

In a partnership between two academic medical centers in Caracas, Venezuela (Luis Razetti School of Medicine at the Universidad Central de Venezuela (UCV)) and New York, USA (New York University (NYU) Grossman School of Medicine), we evaluated the impact of an IM POCUS training program on knowledge and skills of IM physicians at UCV. During 2023-2024, 18 UCV IM physicians participated in the program. The program included online tutorials and quizzes, in-person image interpretation review, and supervised practice. Participants completed a pre-course knowledge assessment, post-course knowledge, skills, and self-confidence assessments, and qualitative feedback regarding course acceptability.

**Results:**

Pre-to-post knowledge assessments demonstrated mean score improvement. Post-course knowledge scores were not significantly different between UCV and NYU cohorts (77% vs. 78%, respectively; p =0.82). Skill scores measured by a hands-on test were comparable between groups, with few significant differences. Learners self-rated increases in confidence during the course, and rated the course as locally acceptable and sustainable.

**Conclusions:**

A standardized, longitudinal, international IM POCUS training program was successfully implemented with faculty learners in a lower-resourced health system, who demonstrated gains in knowledge and skills, and reported high educational value of the partnership. The results support expanding inter-institutional POCUS training programs founded in health equity principles.

## Introduction

Point of care ultrasound (POCUS) is increasingly considered an essential and high-impact tool in internal medicine (IM) for bedside diagnosis and management of urgent medical conditions [1]. Across the United States, 61% of IM residency programs include POCUS training[2], and over half of the medical schools include POCUS educational content [3]. POCUS holds particular value in resource-limited healthcare settings including in low- and middle-income countries [4]. It has documented positive impacts on clinical decision making [5,6], particularly when alternative imaging options can be prohibitively expensive or unavailable.

However, the availability of accessible, longitudinal POCUS training in lower-resourced health systems remains limited [7], reflecting a pervasive theme of inequity in global POCUS education. Barriers to POCUS training include cost-prohibitive equipment, an unmet need for locally relevant curriculum development, and insufficient opportunities for longitudinal supervised practice due to a lack of locally trained faculty and reliance on visiting experts, as brief one-off sessions are not associated with sustainable skilled use [8]. Specifically in Venezuela, a protracted economic crisis has led to a collapsed public healthcare system, mass exodus of healthcare workers, and escalating morbidity and mortality [9]. It is an environment with high potential to benefit from POCUS as a diagnostic and management tool. However, no standardized IM POCUS curriculum at the graduate medical education level currently exists in Venezuela.

A core tension in global medical education partnerships is providing high-quality training in lower-resourced environments while ensuring ethical and sustainable practices. The Brocher Declaration principles, while designed for guiding the development of short-term experiences in global health, are relevant to the design of any international academic partnership.10 These six core principles describe the following aspects: the need for mutual partnership with bidirectional learning; empowering learners to define needs; sustainability and capacity building of program design; complying with ethical and legal standards; respecting all participants; and taking accountability for actions. Alongside these ethical guidelines, the recent successes of technology-enhanced and distance-learning approaches to POCUS training demonstrated that multi-institutional collaboration—as an educational alternative in the face of cost and human resource constraints—can be successful in the design and delivery of curriculum for residents [11], faculty [12], and medical students[13].

We developed an international partnership between two academic medical centers in Venezuela (Luis Razetti School of Medicine at the Universidad Central de Venezuela (UCV)) and the United States (New York University (NYU) Grossman School of Medicine), to conduct and evaluate an IM POCUS training program with faculty learners, based in health equity principles and designed to emulate successful hybrid virtual and in-person curricula. This study aimed to evaluate the impact of the program on UCV learners' POCUS knowledge and skills, in comparison with outcomes from learners of a similar curriculum at NYU, and the local acceptability of the program to participating learners.

## Materials and Methods

### Participants and Setting

In 2023, the need for an IM POCUS training program was expressed by medical eduation leaders at UCV's Luis Razetti Medical School to colleagues at NYU. This was based on understanding that POCUS is increasingly considered a standard component of IM training and, at the time of study initiation, was not available as a formal component of graduate medical education training in Venezuela. All faculty physicians in the IM Service of the Hospital Universitario de Caracas (HUC)—the teaching hospital of UCV's Luis Razetti Medical School—were offered the opportunity to voluntarily participate in the IM POCUS training program, as well as all IM-trained infectious disease specialists in the UCV Tropical Medicine Institute, during the 2023-2024 academic year. Out of 21 eligible IM-trained physicians, 18 chose to participate. Neither remuneration nor incentives were provided to learners for their participation, nor to course developers and implementers. The in-person IM POCUS course instructor at UCV (KOC) had previously received training and certification by the NYU IM POCUS program, whose director (MJ) provided virtual support and supervision throughout the course. The initial training was intentionally conducted with a faculty cohort, rather than residents, in order to first develop departmental leadership and capacity, in advance of the curriculum becoming part of postgraduate training.

### Program Description

The IM POCUS program used in this study was originally developed in alignment with American College of Chest Physicians Critical Care Ultrasound course [14] and included a mix of content both virtual (pre-recorded lectures, online quizzes) and in-person (image interpretation sessions and supervised practice) [12]. The course was considered a standardized example of globally-relevant POCUS competencies for IM physicians, and its content also aligned with recommendations for highest-yield course content in low- and middle-income country settings (left ventricular function assessment; identification and A- and B-lines; identification of pericardial effusion, pleural effusion, and ascites).6 Prior to study initiation, the course material was reviewed and approved by the program director of the IM Residency Program at UCV's Luis Razetti School of Medicine. They requested one additional module beyond the original curriculum for completeness and local epidemiologic relevance, namely the Focused Assessment for Sonography for HIV-associated Tuberculosis protocol. This additional module was taught but not evaluated in the knowledge or skills exams to maintain evaluation standardization across the two sites.

All original course materials were translated into Spanish, and were delivered in biweekly sessions over a 6-month period. After an introductory administrative meeting was held to explain the course curriculum and establish the course schedule, each participant engaged in 10 hours of in-person education. This included six one-hour image interpretation sessions and four one-hour supervised practice sessions, as well as six hours of virtual learning (where participants watched pre-recorded lectures and completed knowledge evaluations, at their convenience, within a specified timeframe and order). The project utilized the Butterfly IQ+ ultrasound device (Burlington, MA) as the core teaching and evaluation tool, of which three devices were donated by the international non-governmental organization “Healing Venezuela.” Additionally, participants were encouraged to use these devices for independent practice between supervised sessions as there was no ultrasound equipment at the hospital available to internists, though this practice time was limited due to three devices being shared by 18 learners.

In-person image interpretation sessions were prepared by MJ at NYU, then translated and and delivered by KOC (hybrid NYU-UCV faculty). KOC supervised the hands-on practice sessions, which were conducted with consenting hospitalized patients who were already under the medical care of the UCV faculty participants, and reported back routinely to MJ to describe program progress and seek complementary virtual guidance. Brocher Declaration principles were built into the course delivery: throughout the course participants were routinely asked for feedback, inputs, and needs, and the course was designed with the intention of sustainability, to build the capacity of faculty who would serve as future course instructors. Given that a majority of online academic POCUS resources are published in English, supplementary, non-NYU, Spanish-language vetted resources were also provided to enhance the curriculum.

### Data Analysis

Course evaluation included developing measures of learners' knowledge, skills, confidence, and programmatic evalution of the course. Specifically, all participants completed a pre-course knowledge assessment immediately prior to course initiation, and a post-course knowledge assessment (26 multiple choice questions testing key principles, image interpretation, and clinical integration) and post-course skills assessment (rating the participant's ability to obtain each of 11 common POCUS views on a scale of 1-3, “1” being poorly done and “3” being well done), in the month after course completion. A single standardized patient was utilized for the skills assessment, with ratings specified by the in-person faculty assessor (KOC). Knowledge and skill scores were compared against the scores of a NYU faculty cohort of learners who participated in a similar course[15] using an independent sample t-test. Similar course features included the use of identical educational materials (aside from language translation). However, notable differences included fewer opportunities for hands-on practice at UCV given fewer available devices, absence of a local simulation center, and lack of resources to organize standardized patient participation. Learners completed a post-course self-confidence assessment comparing perceived confidence at the present moment and prior to the course, along a Likert-scale. Participants also responded to Likert-scaled questions about the utility and acceptability of the course in their existing academic ecosystem, with the option of providing open-ended commentary about the course. Quantitative data were analyzed using Excel for Windows, and brief qualitative comments were coded according to a deductive thematic analysis by KOC, EH, and MC.

### Institutional Review Board, Risk Mitigation, and Reflexivity

The study was designed as a curricular quality improvement initiative and was deemed exempt from the Bioethics Committee of the UCV Facultad de Medicina and the NYU Institutional Review Board. Participant knowledge and skills scores were stored securely and password protected, and were accessible only to KOC who anonymized the data before conducting data analysis. Bias in qualitative data analysis was partially mitigated by the participation of authors holding diverse identities. These included EH and MC as established medical educators in Venezuela, MJ as a leader in IM POCUS education and research in the US, and KOC—who received IM and POCUS training at NYU, facilitated the POCUS course in-person at UCV, and is a graduate from UCV.

## Results

A total of 18 participants completed pre- and post-course evaluations with no participant dropout, and participant demographics are reported in Table 1. UCV course participants were more likely to be mid-career physicians. Few participants had prior POCUS training and only one had used POCUS clinically prior to the course. As illustrated in Table 2, pre-course knowledge scores were identical between the UCV and NYU groups (44/100, p = 0.91), and post-course knowledge scores were similar without statistical significance in the score difference (UCV 77/100 vs. NYU 78/100, p = 0.82). Skills scores were similar between the two learner groups, and most differences were statistically insignificant.

**Table 1.** Demographics of internal medicine (IM) point of care ultrasound (POCUS) course participants from the Luis Razetti School of Medicine at the Universidad Central de Venezuela (UCV) (n=18)

	n	%
**Sex**		
Female	9	50.0%
Male	9	50.0%
**Age**		
< 30	6	33.3%
30-59	10	55.6%
> 60	2	11.1%
**Years since graduation from internal medicine residency**		
0 - 10	6	33.3%
11 - 30	10	55.6%
31 +	2	11.1%
**Additional training (residency or fellowship)**		
Infectious Disease	3	16.7%
**Have you received POCUS training prior to this course?**		
No	12	66.7%
Yes - only online	6	33.3%
Yes - in person or hybrid	0	0.0%
**Did you use POCUS during routine clinical work prior to this course?**		
No	17	94.4%
Yes	1	5.6%

**Table 2.** Comparison of pre- and post-course knowledge and skill assessments (Luis Razetti School of Medicine at the Universidad Central de Venezuela (UCV) vs. New York University (NYU) Grossman School of Medicine)

	UCV (n=18)	NYU (n=21 pre-test; n=17 post-test)	P-value
**Knowledge Evaluation (0-100): mean (SD)**			
Pre-Course Written Exam	44 (0.11)	44 (0.14)	0.91
Post-Course Written Exam	77 (0.14)	78 (0.15)	0.82
Skills Evaluation (1-3)*: mean			
Parasternal long axis	2.4	2.7	0.20
Parasternal short axis	2.8	2.8	1.00
Apical 4 chamber	2.3	2.7	0.08
Subcostal long	2.6	2.5	0.81
Inferior Vena Cava	2.4	2.7	0.37
Lung A-line pattern	2.9	2.9	0.56
Right kidney	2.8	2.7	0.42
Left kidney	2.7	2.7	1.00
Bladder	2.9	2.8	0.25
Aorta short axis	2.9	2.4	0.07
Leg vasculature**	2.6	2.5	0.59

* Test scored on a 3-point scale of (1) Poorly done (2) Partly done (3) Well done.

** Average combined score for common femoral vein above, at, and below the greater saphenous vein, lateral perforator vein, and popliteal vein.

**Figure 1.**
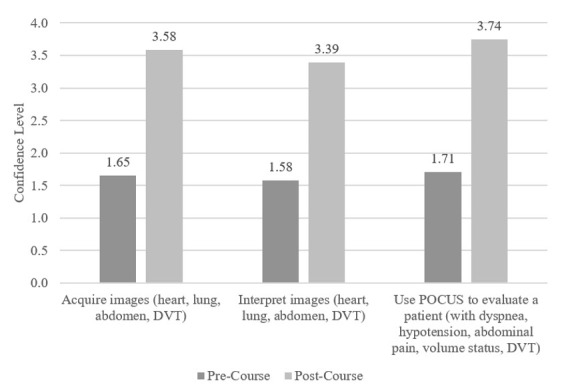
Pre/post course self-rated confidence scores (“How confident are you in your ability to…?”). Differences between each set of pre/post test scores were statistically significant with p-values < 0.0002. DVT = deep vein thrombosis

Figure 1 demonstrates the post-course UCV participant reports, with pre- and post-course confidence levels, in which “1” represented no confidence and “4” represented total confidence. Increases were reported in self-rated ability to acquire images (1.65 to 3.58) and interpret images (1.58 to 3.39), specifically of the heart, lungs, abdomen, and deep veins. Learners also rated increases in their likelihood of using POCUS clinically to evaluate patients with dyspnea, hypotension, abdominal pain, and to rule out a deep vein thrombosis (from 1.71 to 3.74). Differences in pre- and post-course ratings were statistically significant.

Participant perspectives on course evaluation are presented in Table 3. Overall, the course received high ratings for being sustainable, and learners feeling heard and perceiving utility in expanding the course. When provided an open-ended opportunity to submit course feedback, 50% of the comments received focused on items of high value in the course. For instance, comments showed an appreciation for the hands-on supervision (feeling a sense of “continuous accompaniment”), the course structure (“very friendly, both in content and schedule”), the perceived relevance to the existing IM training program (“should be a formal requirement in our residency program”), and the valuable resources provided (“The course ... bibliography (Soni) is excellent.”). The other half of the comments focused on a desire for additional content or changes to the course in future iterations, such as a proportional increase in the amount of hands-on supervised practice sessions, or restructuring of when the hands-on practice occurs (“I would do practical activities accompanied by the teacher, between topics and not just at the end of the theoretical part”). There was also a desire for more advanced content beyond the basics provided (for example, musculo-skeletal POCUS skills and POCUS-guided procedures), technological support (request for more probes and for more guidance in setting-up the required application), and modifications to the written exam evaluation system (namely, to “increase the duration of the clips in the exams since sometimes they are difficult to evaluate”).

**Table 3.** Course evaluation and acceptability, by participants at the Luis Razetti School of Medicine at the Universidad Central de Venezuela (UCV)

Likert-Scaled Statement Agreement (n=18 responses)		**Average Score (1 = strongly disagree; 4 = strongly agree)**
“During this course I felt that my comments and/or concerns were heard and addressed.”		3.9
“The structure of this course seems sustainable for our university environment.”		3.9
“This course would be useful to continue and/or expand at our university.”		3.9
**Open-Ended Comments (n=26 unique comments)**		
***Valued Elements of Course***		***Representative Comment***
In-Person, Hands-On Teaching	27%	"Continuous accompaniment."
Resources Provided	12%	"The course ... bibliography (Soni) is excellent."
High Relevance to Internal Medicine	8%	"POCUS should be a formal requirement in our residency program."
Course Structure	4%	"The program is very friendly, both in content and schedule."
***Desired Additional Content, Changes***		
More Hands-On, Supervised Practice	23%	"I would do practical activities accompanied by the teacher, between topics and not just at the end of the theoretical part."
Additional Content	15%	*Examples*: Musculo-skeletal POCUS, POCUS-assisted procedures, advanced pathologies.
Technology	8%	*Examples*: More probes; More guidance in VPN set-up to download application.
Course Evaluation	4%	"Increase the duration of the clips in the exams since sometimes they are difficult to evaluate."

## Discussion

This IM POCUS training program was designed to demonstrate principles of global health equity in medical education. Faculty learners in this program from a lower-resourced health system increased their knowledge, skills, and confidence, comparable to peers from a higher-resourced health system. While the course itself was curated for a specific country context and a particular inter-institutional partnership, there are several generalizable lessons gleaned from the evaluation for other academic medical institutions interested in global POCUS training programs.

### Learner-Centered, Ethical Curricular Design

Inequalities in global health medical education partnerships are common, structural, persistent, and multi-faceted [16]. Intentional ethical program design is essential, particularly in graduate medical education programming where international investments can be strategic to revitalizing strained health systems[17]. Most essentially, program participants should be included in program design and implementation to ensure the acceptability and utility of the course. Further, creating equitable educational experiences requires additional intervention beyond simply replicating equal experiences, including using pre-course needs assessments to help identify and meet needs specific to an institution. For example, in this course we sought out and included additional, supplementary free Spanish-language educational content given that a majority of free, supplementary POCUS material available online is in English. Additionally, this course was also only possible due to a non-governmental organization partner donation, and the availability of discounted devices through the device-maker's Global Health Program. Device makers and higher-resourced academic programs have the opportunity to promote equity in global POCUS education by partnering to ensure devices are accessible in lower-resourced learner communities. Of note, this need not only applies at an international level, but can also be relevant to providing support to lower-resourced healthcare systems and academic programs within traditionally high-resourced nations like the United States.

### Longitudinal Coursework with In-Person Focus

A longitudinal framework for implementing POCUS education in resource-limited settings can be an essential component of successful curricular design [18]. This course was conducted over several months, and similarly demonstrated notable gains in knowledge and skills among participants. Additionally, a majority of teaching and supervision in this course was conducted in-person. While the course utilized supplementary virtual resources, and while there are virtual elements of POCUS training programs that have demonstrated positive educational impacts (e.g., virtual teaching[19], training [20], and feedback [21]), the in-person presence of a course facilitator likely contributed to a successful learning environment in this study, as reported in qualitative learner feedback. Having a course facilitator available may be a limitation to program adoption by other institutions, if there is not an option for full-time or long-term faculty presence. While the use of digital connective platforms cannot replace in-person instruction, it could be employed to augment existing institutional expertise.

### Bi-Directionality of Educational Value

The focus of this study centered the experiences of and impact on learners in a lower-resourced healthcare system, however, there are notable benefits to higher-resourced academic partners becoming involved in global health initiatives [22]. The next expanded steps of the program evaluated in this manuscript include participation in inter-institutional case conferences to share POCUS utility in the diagnosis and management of pathophysiology that may be more common in one locale yet relevant to others. For example, the Focused Assessment for Sonography for HIV-associated Tuberculosis protocol, or the use of POCUS to quickly diagnose a liver abscess caused by Entamoeba histolytica, are POCUS-uses more clinically relevant in Venezuela, and the expertise that we anticipate will be developed in these skills can be shared with colleagues internationally. It is important for higher-resourced institutions to consider their gains from participation in global health POCUS education and research partnerships in alignment with ethical standards. For example, considering one's impact on the local setting, the potentially disruptive effects of one's presence, and the navigation of power dynamics to achieve respectful collaboration [23].

### Limitations

The small sample size of our cohorts limits the generalizability of the study. The program was possible only due to donations of time—by course developers and supervisors, and equipment —which may not be feasible in all settings. Ideally, those who design and deliver medical education—especially those from lower-resourced healthcare systems—will be compensated for their work. There is also limited understanding of the course's impact on knowledge and skills given the only assessment was completed immediately after the course concluded, and not in a longitudinal manner. Additionally, challenges with the implementation of this course were predominantly related to the availability of devices for independent practice and the lack of education resources to standardize learning experiences; namely lacking a simulation center, specialized manequins, and standardized patients.

### Next Steps

Based on feedback from our initial training program, beginning in 2026 UCV's IM Residency curriculum will formally include IM POCUS training as a competency requirement. In addition to the HUC, there are six teaching hospitals with IM programs affiliated with UCV, and our funding partner has procured additional ultrasound devices to begin program expansion to additional UCV teaching hospitals. At HUC, IM residents are already participating (since 2024) in the IM POCUS training. The original cohort of UCV faculty learners are involved with in-person resident teaching, as well as the creation of updated educational materials. We are also in conversation with The Venezuelan Society of Internal Medicine—the leading national professional society for the speciality—about creating an IM POCUS certification standard aligned with existing international guidelines, so that any course graduate who has earned a certificate of course completion may additionally earn a validated certification, including expert review of individual portfolios.

Additional next steps of this project include a routine, online inter-institutional conference series between NYU and UCV for sharing clinical cases in which POCUS aided diagnosis or management, to augment the bidirectionality of learning between academic centers. We also plan to continue studying our initial cohort and future cohorts, to better understand knowledge and skill retention over time, and to research the impact of POCUS training on patient outcomes. For example, we are currently studying the diagnostic precision of POCUS to identify deep venous thrombosis in hospitalized patients, compared to a formal vascular study. Research is also being considered to measure the cost-savings of the POCUS program, and if skilled use improves diagnostic and management decisions.

## Conclusion

This evaluation of an IM POCUS training program suggests that a course already certified in the United States was translatable to an international, lower-resourced setting. Learners achieved similar rates of success, and rated themselves as having grown in confidence during the course. The program was considered ethically acceptable as a sustainable curricular offering. It is essential to design interinstitutional academic partnerships that focus on equity principles: commitment to capacity-building; identification of bidirectional educational value for learners from both settings; and empowerment of participants to guide curricular development in defining needs and direction.

## Data Availability

The datasets used and analyzed during the current study are available from the corresponding author on reasonable request.
